# An Image Stereo Matching Algorithm with Multi-Spectral Attention Mechanism

**DOI:** 10.3390/s23198179

**Published:** 2023-09-29

**Authors:** Zhenhua Quan, Bin Wu, Liang Luo

**Affiliations:** 1 Institute of Electronic Engineering, China Academy of Engineering Physics, Mianyang 621900, China; 2 School of Information Engineering, Southwest University of Science and Technology, Mianyang 621000, China; wubin@swust.edu.cn (B.W.); luoliang@swust.edu.cn (L.L.)

**Keywords:** stereo matching, deep learning, attention mechanism

## Abstract

With the advancement of artificial intelligence technology and computer hardware, the stereo matching algorithm has been widely researched and applied in the field of image processing. In scenarios such as robot navigation and autonomous driving, stereo matching algorithms are used to assist robots in acquiring depth information about the surrounding environment, thereby improving the robot’s ability for autonomous navigation during self-driving. In this paper, we address the issue of low matching accuracy of stereo matching algorithms in specular regions of images and propose a multi-attention-based stereo matching algorithm called MANet. The proposed algorithm embeds a multi-spectral attention module into the residual feature-extraction network of the PSMNet algorithm. It utilizes different 2D discrete cosine transforms to extract frequency-specific feature information, providing rich and effective features for cost computation in matching. The pyramid pooling module incorporates a coordinated attention mechanism, which not only maintains long-range dependencies with directional awareness but also captures more positional information during the pooling process, thereby enhancing the network’s representational capacity. The MANet algorithm was evaluated on three major benchmark datasets, namely, SceneFlow, KITTI2015, and KITTI2012, and compared with relevant algorithms. Experimental results demonstrated that the MANet algorithm achieved higher accuracy in predicting disparities and exhibited stronger robustness against specular reflections, enabling more accurate disparity prediction in specular regions.

## 1. Introduction

The binocular stereo vision system captures images using a pair of cameras. The frame rate of the cameras is selected based on the speed of the scene, and high- and low-resolution images are acquired accordingly. Preprocessing techniques such as filtering are applied to the images before performing stereo matching [1]. Stereo matching utilizes the disparity information obtained from the differences between the two images to calculate depth and other related information. With the advancements in artificial intelligence technology and computer hardware, stereo matching algorithms have been extensively researched and applied. In scenarios such as robot navigation [2] or autonomous driving [3], stereo matching algorithms can assist robots in obtaining depth information about the surrounding environment, thereby enhancing their autonomous navigation capabilities.

In 2002, Scharstein et al. [4] proposed a four-step framework for stereo matching, which includes cost computation, cost aggregation, disparity calculation, and disparity optimization. This framework has been widely adopted and remains in use to this day. Traditional stereo matching algorithms can be categorized into local matching, global matching, and semi-global matching based on the pixel range they process. Local matching algorithms compare a small neighborhood around each pixel in one image with the corresponding region in the other image. The most similar region is identified, and its center point is considered as the matching point. Global matching algorithms compute a cost map between a pair of stereo images and use techniques such as dynamic programming or energy-based algorithms to find an optimal path in the cost map, which represents the matching point of each pixel in the other image, resulting in a disparity map. On the other hand, semi-global matching algorithms, which are computationally efficient, calculate the cost between each pixel in the stereo image pair and all possible matching points in the other image. Cost aggregation techniques are then applied to aggregate the costs in four directions, resulting in the cost sum for each pixel in each direction. Finally, the disparity map is computed based on these cost sums. However, these traditional stereo matching algorithms suffer from limitations such as poor performance in complex scenes, slow computation speed, and low matching accuracy.

With the improvement of computing power and the increase in data volume, convolutional neural networks have brought more possibilities for solving the problem of stereo matching and have gradually become a research focus [5]. Deep learning techniques optimize algorithm performance through a large amount of image data [6], achieving optimal results by autonomously learning and optimizing representations, thereby improving the accuracy and robustness of stereo matching. The stereo matching algorithm based on deep learning extracts multiple features through multi-layer convolution for cost computation and uses regularization methods for cost aggregation to optimize the cost volume, thereby reducing the mismatch rate and improving the matching speed.

**Figure 1:**
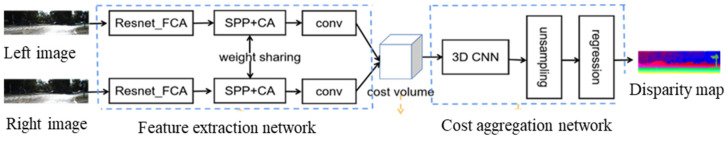
MANet network architecture.

In the process of stereo matching in practical scenarios, factors such as occlusion, lighting, and weak texture can affect the matching accuracy between the left and right images, leading to higher mismatch rates in certain pathological regions. Attention mechanisms have been widely used or improved in various computer vision tasks in recent years. This paper introduces multiple attention mechanisms into the feature-extraction network of stereo matching algorithms, assigning different weights to features from the perspectives of frequency and position, reducing the impact of lighting conditions, and better utilizing feature information to accomplish stereo matching tasks. To address the high mismatch rate in pathological regions such as reflections in binocular stereo matching, a multi-attention-based stereo matching algorithm called MANet was proposed by introducing multiple attention mechanisms into the feature-extraction stage of the PSMNet algorithm. The algorithm focuses more on the important parts of different features, enabling the extraction of useful feature information in a more precise manner. The main improvements include:

(1) Introducing a multi-spectral attention mechanism to weight different frequency feature maps, helping the residual network capture various detailed features of the input images and improve recognition accuracy in complex scenes.
(2) Introducing a coordinated attention mechanism in the pyramid pooling module to extract spatial features at different scales. By adaptively learning the positional relationships of features at each scale, the feature-extraction capability is enhanced. The extracted features from the left and right images are concatenated using a shift-and-stitch method to construct a four-dimensional cost volume, which is then optimized and used for disparity calculation through stacked hourglass networks. The network architecture is illustrated in Figure 1.

## 2. Related Work

A mathematical model is constructed for the global algorithm to build an energy function, utilizing methods such as Newton’s method and gradient descent to minimize the energy function and find the optimal matching solution. Shahbazi et al. [7] from the University of Calgary employed the non-parametric census transform as the data term and used the geometric features of intrinsic curves as the smoothness term to minimize the energy function, thereby improving the problem of a high mismatch rate caused by image occlusion. Zhou Jiali et al. [8] from Zhejiang University of Technology based their approach on conventional graph cut algorithms. They corrected the matching region based on the labels of the matching blocks and spatial geometric information, continuously updating the selected labels to find the labels that minimize the global energy. By applying the left–right consistency criterion and mean filtering to refine the disparity map, they achieved higher sub-pixel-level accuracy in the matching disparity. The matching accuracy in the edge and occlusion regions of the image pair was significantly improved.

The matching cost convolutional neural network (MC-CNN) was proposed by Zbontar et al. [9] of the University of Ljubljana. In the cost-computation stage, a convolutional neural network is used to train on input image patches and annotated images, obtaining matching cost values. These values are then optimized using a cross-cost aggregation method, incorporating a left–right consistency check and bilateral filtering for refining the disparity map. Seki et al. [10] from Toshiba Corporation introduced the SGMNet algorithm, which utilizes a convolutional neural network to automatically learn penalty parameters, eliminating the manual adjustment process in traditional SGM algorithms. This algorithm divides the disparity transition along the scanline into positive and negative disparity transitions based on different occlusion relationships between objects, ensuring good disparity prediction even in pathological regions. However, non-end-to-end stereo matching algorithms often fail to meet practical requirements due to their high time complexity. They remain within the traditional stereo matching framework and require significant time and effort for parameter adjustments, such as filter size and matching window size.

The end-to-end stereo matching algorithm learns the features in the input images adaptively through a deep learning model, eliminating the need for manual feature design and selection. The model is more adaptable to different scenes and details. Mayer et al. [11] constructed a virtual synthetic dataset and proposed the end-to-end network DispNetC. This network introduced an autoencoder–decoder structure, taking in left and right images and outputting a disparity map without any post-processing steps. Xu et al. [12] from the University of Science and Technology of China extracted features of different resolutions using a shared feature pyramid network. They designed three same-scale aggregation modules to optimize different resolution features and proposed the adaptive aggregation network (AANet), which fuses features through a cross-scale aggregation module to address the issues of large parameter and computational requirements in deep stereo matching networks, thereby improving algorithm efficiency. Vladimir et al. [13] from Google introduced HITNet, a convolutional neural network for real-time stereo matching. It infers disparity through fast multi-resolution initialization and transformation, without incurring significant costs. By propagating information across levels, it reduces algorithm complexity while improving matching accuracy. Tang Haifeng et al. [14] from Inner Mongolia University proposed DFFNet, an end-to-end stereo matching network that incorporates dense feature fusion. They utilized multiple residual modules to construct a feature pyramid network, capturing multi-scale contextual information with a low parameter count. The network enhanced its matching capability in complex regions such as weak texture areas and edges through dense fusion modules and mixed attention modules, thereby improving the extraction of useful information.

This paper proposes a novel solution for the high mismatch rate in stereo matching caused by factors such as occlusion, lighting, and weak texture in real-world scenarios. To address this issue, we employed an end-to-end stereo matching algorithm based on deep learning. By utilizing convolutional neural networks to extract different features from global information and incorporating attention mechanisms, we designed a deep neural network to reduce the mismatch rate in pathological regions, such as reflective and edge areas. This approach offers a new solution for handling pathological image processing in stereo matching algorithms.

## 3. Proposed Method

### 3.1. Multi-Attention Feature-Extraction Network

#### 3.1.1. Residual Network with Embedded Multi-Spectral Attention Mechanism

**Figure 2:**
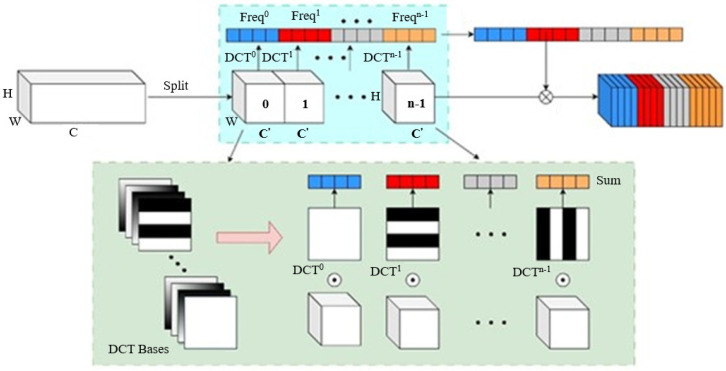
Multi-spectral attention mechanism network architecture.

Qin et al. [15] proposed a frequency channel attention network called FcaNet, which differs from the commonly used channel attention method, SENet, in that it does not solely rely on global average pooling. Instead, this pooling method is used as a frequency feature-extraction technique, employing different two-dimensional discrete cosine transform (DCT) pooling methods for different frequency components. The feasibility of this approach was mathematically derived and proven by the authors, and its effectiveness was demonstrated through image classification experiments. For this paper, we applied this attention mechanism to the stereo matching algorithm, forming a multi-spectral attention module. The network structure is illustrated in Figure 2.

The input features are denoted as X, with a channel number of C and a size of H×W. The channel number C is divisible by n, and the input is divided into n feature maps. Each feature map is multiplied by different two-dimensional discrete cosine transform frequency components, which can be understood as different pooling methods. By introducing more frequency components, different channel features can be extracted. The resulting compressed results can be used as channel attention.

(1) $$Freq^{i}=2DDCT^{ui,vi}(x^{i}),i\in \{0,1,\cdots ,n-1\}$$

where [ui,vi] is the frequency component index corresponding to input $X^{i}$, the frequency components of each part are concatenated, and the final output multi-spectral vector is obtained through activation sigmoid. The multi-spectral vector is then used for learning in the fully connected layer to obtain the attention feature map.

(2) $$att=sigmoid(f_{c}(cat(Freq^{0},Freq^{1}\cdots ,Freq^{n-1})))$$

where cat represents the concatenation operation and $f_{c}$ denotes the fully connected layer.

**Figure 3:**
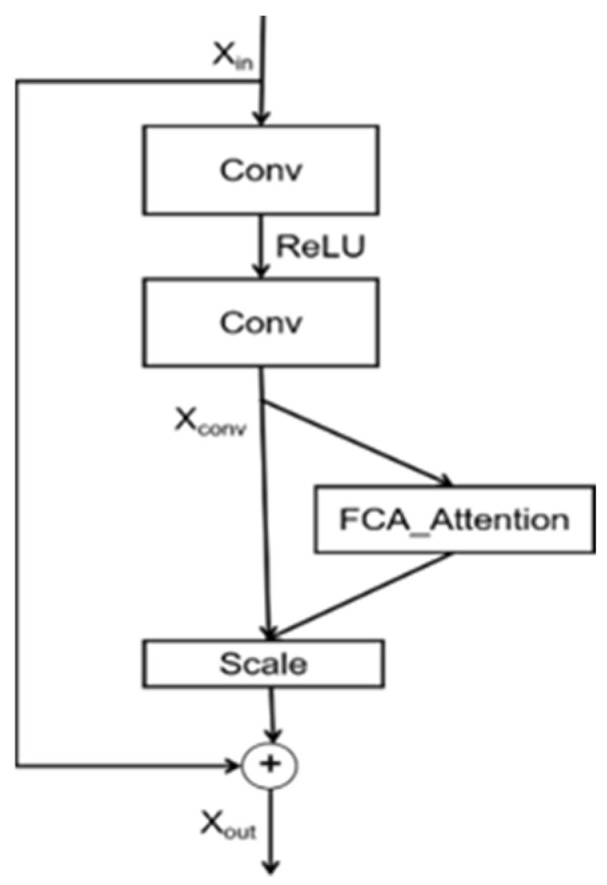
Residual network with embedded multi-spectral attention mechanism.

The residual network, ResNet, employs skip connections to increase the depth of the network, enabling more effective feature extraction and avoiding the problem of gradient vanishing. Multi-spectral attention treats global average pooling as a two-dimensional frequency component, extracting effective features through the frequency dimension. For this paper, we embedded the multi-spectral attention mechanism within the residual blocks of the ResNet, allowing for better extraction of various features from input images by capturing different frequency feature information. The network architecture is illustrated in Figure 3.

#### 3.1.2. Spatial Pyramid Pooling Module with Embedded Coordinated Attention Mechanism

**Figure 4:**
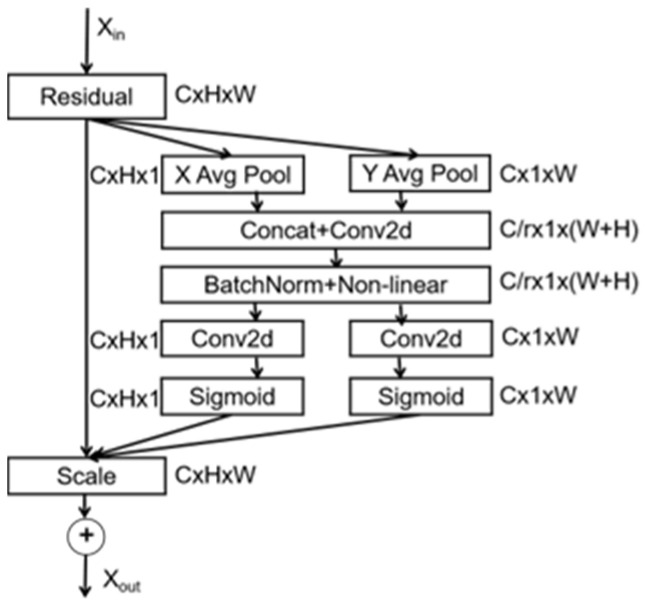
Illustration of the architecture of the coordinated attention network.

The channel attention mechanism focuses solely on the channel information of the network, which can improve performance to some extent, but it does not consider the influence of positional features. Hou et al. [16] proposed a new coordinated attention network (CA) that outperforms the channel attention SENet in classification tasks. The network structure is shown in Figure 4.

The coordinated attention mechanism utilizes average pooling along the width (W) and height (H) dimensions of the input image to perform one-dimensional feature encoding, resulting in feature maps $z^{h}$ and $z^{w}$. These two feature maps are then concatenated, and batch normalization is applied to ensure that the input data follow a distribution with zero mean and unit variance. Scaling and offset parameters are adjusted accordingly. Finally, the sigmoid function is used to generate new features.

(3) $$f=\sigma (F_{1}([z^{h},z^{w}]))$$

In the equation, [ , ] denotes the concatenation of two spatial dimension feature encoding results and $F_{1}$ represents a shared 1×1 convolution operation. The new feature map is decomposed into tensors $f^{h}$ and $f^{w}$ along the width W and height H dimensions, respectively.

(4) $$\begin{array}{cc} g^{h} & =\sigma (F_{h}(f^{h}))\\ g^{w} & =\sigma (F_{w}(f^{w})) \end{array}$$

In this expression, σ represents the sigmoid function, while $F_{h}$ and H are two distinct 1×1 convolutional transformation functions. By applying an activation function, tensors $f^{h}$ and $f^{w}$ are transformed into attention weights $g^{h}$ and $g^{w}$, respectively. The expression that coordinates attention is as follows:

(5) $$y_{c}(i,j)=x_{c}(i,j)\times g_{c}^{h}(i)\times g_{c}^{w}(i)$$

**Figure 5:**
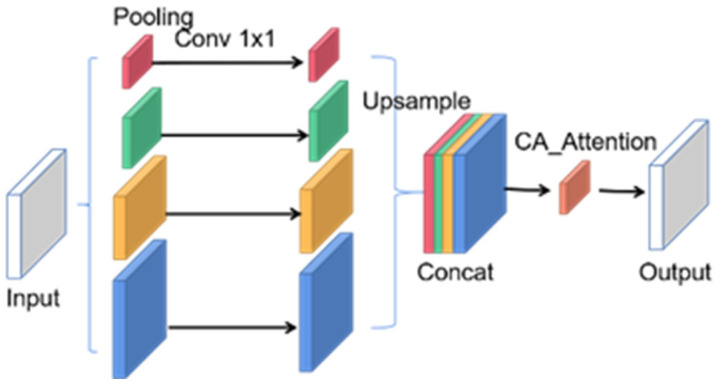
Depiction of the structure of the embedded coordinated attention spatial pyramid pooling network.

This work embedded the co-attention mechanism into the pyramid pooling module to preserve positional relationship information between features. The network architecture is illustrated in Figure 5.

The initial operation of the embedded coordinated attention spatial pyramid pooling (ECASP) network structure is like the pyramid pooling module, where different average pooling methods are used to extract four feature vectors of varying spatial sizes. These feature vectors of different scales are then resized to the same size and concatenated to form a new feature vector, which is input into the coordinated attention mechanism. The coordinated attention mechanism encodes the corresponding weights for both the width and height directions and multiplies them with the original input feature map, resulting in a feature map with positional information.

### 3.2. Cost Structure Construction

In constructing the cost volume, the left and right images are processed using a Siamese network for feature extraction, resulting in two feature tensors for the left and right images. These two feature tensors are then concatenated using a shift operation to construct a 4D cost volume (height × width × disparity × feature size). Compared to the dot product and distance-based methods, this approach effectively preserves the feature dimensions in the generated cost volume, thereby reducing matching errors.

(6) V=W×H×D×2C

Here, V represents the cost volume constructed, while W, H, D, and C, respectively, denote the width, height, maximum candidate disparity, and number of feature channels. The left and right feature maps have a size of W×H×C, and their C channels are horizontally shifted starting from the 0th pixel until reaching D−1, generating feature tensors of size W×H×D×C. These two feature tensors are then concatenated correspondingly to obtain a 4D matching cost volume.

### 3.3. Cost Aggregation Network

**Figure 6:**
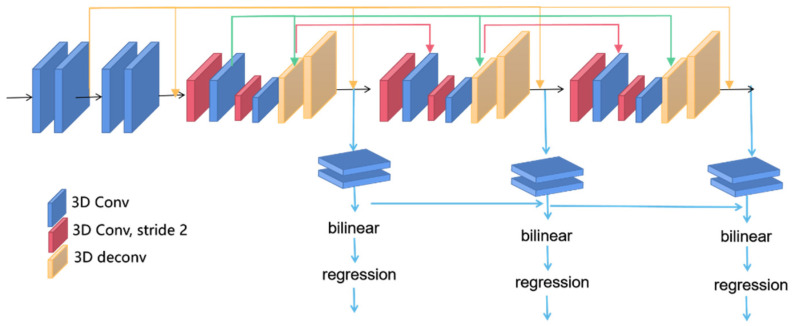
Depiction of a 3D stacked hourglass network diagram.

After constructing the cost volume, regularization processing is required, similar to the cost aggregation step in traditional stereo matching algorithms. By utilizing the contextual information from cost volumes at different scales, the quality of the cost volume is optimized. Since the constructed 4D cost volume requires training with 3D convolutional layers, it is prone to high computational complexity. Therefore, for this paper, we adopted the same cost aggregation network as PSMNet [17], which has an encoding–decoding structure that effectively addresses the issue of computational complexity and accomplishes dense prediction tasks. The network structure is illustrated in Figure 6.

The three-dimensional stacked hourglass structure consists of three hourglass networks. Convolution is applied to the constructed 4D cost volume, using convolution with a stride of 2 for encoding to reduce the feature size. Then, decoding is performed using deconvolution to restore it to the original feature size. This approach effectively utilizes contextual information, and the feature maps of different hourglass networks are connected through different network layers, enabling the comprehensive utilization of cost volumes at different depths and global contextual information. Each hourglass network generates a disparity map using trilinear interpolation and regression. The final disparity map is obtained by weighting and summing the three output disparity maps. During the training phase, the weighting coefficients are set to 0.5, 0.7, and 1, while during the testing phase, the coefficients are set to 0, 0, and 1. The disparity calculation method and loss function for regression are described in Section 4.

### 3.4. Visual Disparity Calculation

In the visual disparity calculation section, the soft algorithm is employed to obtain the probability of each disparity for every pixel within the maximum disparity range. The expression is as follows:

(7) $$p_{d}=softmax(x_{i})=\frac{e^{x_{i}}}{\sum_{j=0}^{J}e^{x_{i}}}$$

In the equation, $p_{d}$ represents the probability of the true value disparity map.

To obtain the predicted disparity value of a pixel, multiply the disparity value of each pixel by its estimated probability and sum them up.

(8) $$d_{pre}=\sum_{d=0}^{D_{\max}}d*p_{d}$$

Here, $d_{pre}$ represents the predicted disparity value and $D_{max}$ is the maximum disparity value.

The stereo matching algorithm used for this paper adopted the L1 loss function to train the network, which is expressed as follows:

(9) $$L\left(d,d_{pre}\right)=\frac{1}{N}\sum_{i=1}^{N}{\mathrm{smooth}}_{L_{1}}\left(d_{i}-d_{pre}\right)$$

In the equation, N represents the total number of labeled pixels in the feature map.

(10) $${\mathrm{smooth}}_{L_{1}}(x)=\left\{\begin{array}{ll} 0.5x^{2}, & \;\mathrm{if}\;|x|<1\\ |x|-0.5, & \;\mathrm{otherwise}\; \end{array},\right.$$

## 4. Experimental Results and Analysis

To evaluate the predicted disparity performance of the proposed multi-attention stereo matching algorithm, MANet, the dataset and experimental setup are introduced in Section 4.1. Evaluation metrics are presented in Section 4.2. In Section 4.3, experiments are presented on the SceneFlow and KITTI image datasets to compare MANet with other stereo matching algorithms, including GCNet [18], SegStereo [19], DispNetC [11], AANet [12], DFFNet [14], CRL [20], Bi3D [21], and PSMNet [17].

### 4.1. Dataset and Experimental Parameters

#### 4.1.1. Dataset

**Figure 7:**
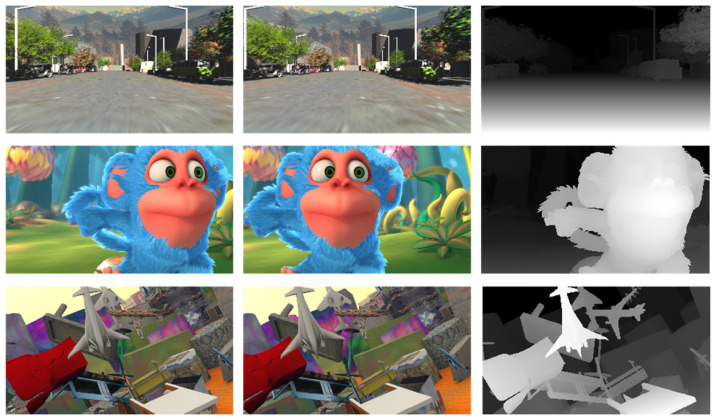
SceneFlow dataset example.

**Figure 8:**
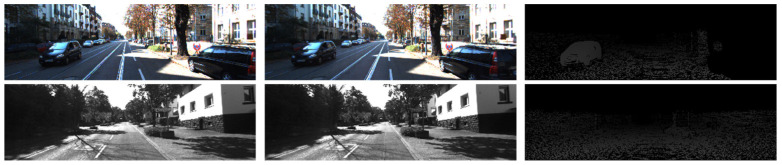
KITTI dataset example.

(1) SceneFlow dataset: A virtual synthetic dataset. It consists of training and testing sets from three different scenes. The FlyingThings3D dataset contains complex textured backgrounds with dense disparity maps for supervised learning. The Monkaa dataset mostly consists of images with weak or repetitive textures. The Driving dataset simulates images of urban roadside scenes. All images have a size of 540 × 960, and there are a total of 39,824 stereo image pairs with a maximum disparity of 192. This dataset is commonly used for pre-training stereo matching network models (Figure 7).

(2) KITTI dataset: It is a widely used stereo matching dataset consisting of real-world, captured images of autonomous driving scenes. The KITTI2015 dataset has image dimensions of 1242 × 375, with 200 pairs of images for training and testing. During network training, 4/5 of the image pairs are used for the training set, while 1/5 of the image pairs are used for validation. The KITTI2012 dataset has image dimensions of 1226 × 370 and includes 194 pairs of training images and 195 pairs of testing images. Figure 8 shows two pairs of images from the KITTI dataset, including the left and right images of the stereo image pairs, as well as the corresponding ground truth disparity maps.

#### 4.1.2. Experimental Environment and Parameter Settings

The proposed algorithm was implemented using the PyTorch deep learning framework and trained and tested on a single NVIDIA 3070Ti graphics card. Initially, training was conducted on the SceneFlow dataset, where input images of size 540 × 960 were randomly cropped to 256 × 512. The learning rate was set to 0.001, and the model was trained for 16 epochs using the Adam optimizer. The entire dataset was used for training from scratch, which took approximately 50 h. Subsequently, training was performed on the KITTI2015 dataset for 1000 epochs. The learning rate was set to 0.001 for the first 200 epochs and then decayed to 0.0001. This training process took around 60 h. Finally, the KITTI2012 dataset was used for training for 1000 epochs, with the same batch size, optimizer, learning rate settings, and training approach as the KITTI2015 dataset.

### 4.2. Algorithm Evaluation Metrics

An evaluation of stereo matching test results from both quantitative and qualitative aspects was performed. Qualitative evaluation involves visually comparing the predicted disparity maps of various algorithms, such as disparity prediction performance in image edge regions, reflective regions, and occluded regions. Quantitative evaluation is conducted using two metrics: endpoint error and mismatch rate.

SceneFlow dataset employs endpoint error as an evaluation metric, which can be represented by training and testing a network model to obtain predicted disparity maps. The specific implementation involves accumulating the disparity differences between the disparity values of individual pixels in the image and their corresponding ground truth disparities, and then dividing this sum by the total number of labeled pixels in the image.

(11) $$EPE=\frac{1}{N}\sum_{i=1}^{N}\left|D_{pre}(i)-D_{GT}(i)\right|$$

The KITTI dataset is commonly evaluated using the outlier percentage as a performance metric. This metric represents the percentage of pixels in the predicted disparity map, based on either the left or right reference image, that have an absolute error greater than a certain threshold. The threshold, denoted as X, can be set to 2, 3, or 5.

(12) $$PE=\frac{1}{N}\sum_{i=1}^{N}\left[\left|D_{pre}(i)-D_{GT}(i)\right|\geq x\right]$$

$D_{pre}(i)$ represents the predicted disparity value of a pixel in the test image, $D_{GT}(i)$ represents the ground truth disparity value of a pixel in the test image, and N represents the total number of labeled pixels in the test image.

### 4.3. Experimental Analysis

#### 4.3.1. Ablation Experiment

We performed a comparison of the improved feature-extraction network module of the proposed algorithm with the baseline feature-extraction network of the PSMnet network. The evaluation metrics on the KITTI2015 test set and SceneFlow test set were the three-pixel mismatch rate and the mixed endpoint error. The experimental results are shown in Table 1.

The ResNet_FCA module in Table 1 introduced a multi-spectral attention mechanism into the residual network, while the SPP_CA module incorporated a coordinated attention mechanism into the spatial pyramid pooling. In the ablation experiments, the SPP_CA network as well as the combination of the ResNet_FCA and SPP_CA modules were compared with the original feature-extraction network ResNet + SPP. The spatial pyramid pooling SPP_CA, embedded with the coordinated attention mechanism, reduced the endpoint error to 1.083 px on the SceneFlow dataset and achieved a three-pixel mismatch rate of 1.92% on the KITTI2015 dataset. The ResNet_CA + SPP_FCA network, which incorporates multiple attention mechanisms, reduced the endpoint error to 1.072 px and achieved a three-pixel mismatch rate of 1.86%. Compared to the baseline network, these improvements represented reductions of 1.65% and 6.06%, respectively, demonstrating the effectiveness of introducing multiple attention mechanisms to enhance feature-extraction performance in the module.

#### 4.3.2. Comparative Experimental Analysis on Three Major Public Datasets

(1) The quantitative comparison results on the SceneFlow dataset, using the endpoint error as the evaluation metric, are presented in Table 2.

Comparisons were made between the proposed method and other methods, including AANet [12], GCNet [18], DispNetC [11], and PSMNet [17], on the SceneFlow dataset. The quantitative evaluation results are shown in Table 2, where “px” represents pixels. The proposed algorithm achieved lower endpoint error (EPE) values than the other four algorithms on the SceneFlow dataset. Specifically, compared to AANet, the EPE was reduced by 0.358; compared to GCNet, the EPE was reduced by 1.438; and compared to DispNetC, the EPE was reduced by 0.608. Compared to the baseline PSMNet algorithm, the EPE was reduced by 0.02 and the endpoint error was reduced by 1.65%.

**Figure 9:**
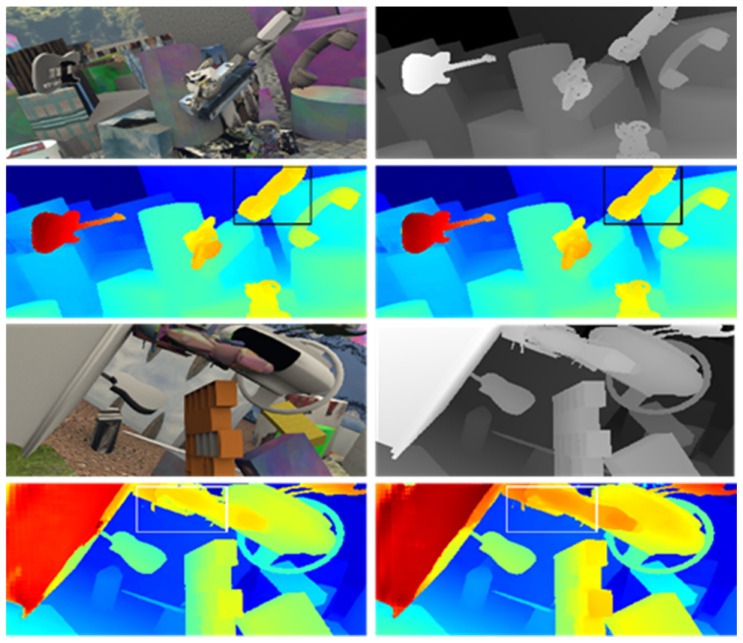
Comparison of disparity prediction on SceneFlow dataset.

(2) Qualitative comparison results in the SceneFlow test dataset (with disparity prediction as the evaluation metric) are shown in Figure 9.

In the experiment, two pairs of test images from the SceneFlow dataset were selected for stereo disparity prediction. The top-left image represents the left image of the stereo image pair, the top-right image represents the ground truth disparity map, the bottom-left image represents the disparity map predicted by the PSMNet algorithm, and the bottom-right image represents the disparity map predicted by the MANet algorithm. From the results of the telephone and car body unit image processing shown in the figures, it can be observed that MANet achieved a more accurate disparity prediction compared to the PSMNet algorithm by assigning different weights to the features.

(3) The quantitative comparison results with other methods submitted to the KITTI leaderboard website on the KITTI2015 test dataset, using pixel error rate as the evaluation metric, are shown in Table 3.

Table 3 presents the quantitative evaluation results of the proposed algorithm in this paper on the KITTI2015 dataset, compared to DispNetC [11], GCNet [18], CRL [20], Bi3D [21], DFFNet [14], AANet [12], and PSMNet [17]. The evaluation metrics D values are computed for each algorithm in the background region (bg), foreground region (fg), and overall region (all). From Table 3, it can be observed that the proposed algorithm achieved lower D1-bg, D1-fg, and D1-all values compared to the other methods in all regions and non-occluded regions. Compared to the baseline PSMNet algorithm, the proposed algorithm reduced the pixel mismatch rate by 4.31% in all regions and by 5.14% in all pixels of non-occluded images (NOC).

**Figure 10:**
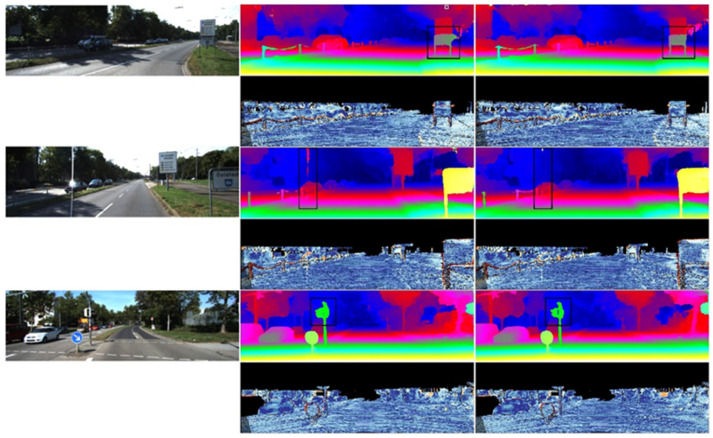
Comparative analysis of predicted disparity maps and error maps on KITTI2015 dataset.

(4) The qualitative comparison results on the KITTI2015 test dataset (with disparity prediction as the evaluation metric) are shown in Figure 10.

In this study, three pairs of test images were selected from the KITTI2015 dataset for the purpose of evaluating and comparing the performance of stereo disparity prediction. The top-left image represents the left image of a stereo image pair, while the middle image displays the disparity map predicted by the PSMNet algorithm. On the right side, the disparity map predicted by our proposed algorithm, MANet, is shown. The corresponding error maps are displayed below each disparity map. From the images, it can be observed that the disparity maps predicted by the MANet algorithm exhibited superior performance. This is evident from the clear visibility of the license plates on the roadside and the traffic lights in the middle of the road. Furthermore, in the second image, it can be observed that the MANet algorithm effectively reduced the interference caused by reflections from vehicles on the road, resulting in accurate disparity prediction.

(5) The quantitative comparison results with other methods submitted to the KITTI leaderboard website on the KITTI2012 test dataset are presented in Table 4, using pixel error rate as the evaluation metric.

Table 4 presents the quantitative evaluation results of our algorithm on the KITTI2015 dataset compared to SGMNet [10], DispNetC [11], GCNet [18], SegStereo [19], AANet [12], and PSMNet [17]. From Table 4, it can be observed that the MANet algorithm achieved a mismatch rate of 2.26% and 2.87% for non-occluded regions and the entire region, respectively, at an error threshold of 2. These values represent a reduction of 7.37% and 4.65% compared to the baseline PSMNet algorithm. As the error threshold increased to 3 and 5, the reduction in mismatch rate became more significant, indicating an overall decrease in mismatch rate.

**Figure 11:**
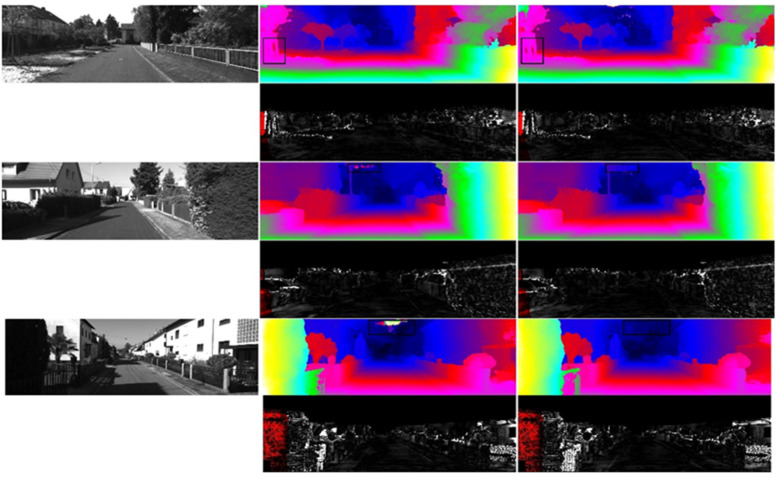
Comparison of predicted disparity maps and error maps on KITTI2012 dataset.

(6) The qualitative comparison results on the KITTI2012 test dataset, using disparity prediction as the evaluation metric, are presented in Figure 11.

From the figure, it can be observed that, compared to the PSMNet algorithm, the MANet algorithm predicted more continuous disparities in areas such as the sky and trees.

(7) The quantitative comparison results with other methods submitted to the KITTI leaderboard website on the KITTI2012 test dataset, specifically for the evaluation of reflective regions, are presented in Table 4, using pixel error rate as the evaluation metric.

From Table 5, it can be observed that the MANet algorithm achieved a significant reduction in error rate compared to other algorithms in recognizing reflective regions in the KITTI2012 dataset. For instance, when the error threshold was set to 5, the pixel error rate in the non-occluded and overall regions was reduced by 21.18% and 19.43%, respectively, compared to the baseline PSMNet algorithm. Furthermore, when compared to the ACVNet algorithm, the reduction in pixel error rate was 12.80% and 8.65% in the respective regions.

## 5. Conclusions

This article proposes a multi-attention-based stereo matching algorithm called MANet. The algorithm introduced a multi-attention mechanism in the feature-extraction network (residual network) to extract various details and features of the input image by weighting the feature maps from different layers. A coordinated attention mechanism was introduced in the pyramid pooling module to capture positional relationship information between features and enhance feature extraction. Experimental tests were conducted on three public datasets: SceneFlow, KITTI2015, and KITTI2012. The results were compared with existing popular stereo matching algorithms such as GCNet, SegStereo, DispNetC, AANet, DFFNet, CRL, Bi3D, and PSMNet. The experimental results demonstrated that the multi-attention and coordinated attention mechanisms complement each other, playing a positive role in weight correction and error backpropagation throughout the network. The MANet algorithm effectively identified salient features of different objects by aggregating rich matching information, extracted more comprehensive and effective features, reduced matching errors, and achieved higher predictive disparity accuracy. The algorithm exhibited strong robustness against specular reflections, accurately predicted disparities in specular reflection pathological regions, and sensitively identified salient features of objects in these regions. This algorithm can be used for tasks such as image segmentation, object detection, and image enhancement. By analyzing salient features in regions affected by reflections, it is possible to extract the contours and characteristics of the main subject, thereby achieving effective processing of the image. Future research will focus on improving the algorithm’s computational speed and performance in other challenging scenarios, such as estimating accuracy in occluded areas and regions with weak textures.

## Figures and Tables

**Table 1:** Experimental results of different feature-extraction network modules.

Network Setting	KITTI2015	Scene Flow
-	Val Err (%)	End Point Err (px)
ResNet + SPP	1.98	1.09
ResNet + SPP_CA	1.92	1.083
ResNet_FCA + SPP_CA	1.86	1.072

**Table 2:** Comparative test results on SceneFlow dataset.

Algorithm	MANet	AANet [12]	GCNet [18]	DispNetC [11]	PSMNet [17]
EPE (px)	1.072	1.43	2.51	1.68	1.09

**Table 3:** Comparison of results on KITTI2015 test dataset.

	All (%)			Noc (%)		
Algorithm	D1-bg	D1-fg	D1-all	D1-bg	D1-fg	D1-all
DispNetC [11]	4.32	4.41	4.34	4.11	3.72	4.05
GCNet [18]	2.21	6.16	2.87	2.02	5.58	2.61
CRL [20]	2.48	3.59	2.67	2.32	3.12	2.45
Bi3D [21]	1.95	3.48	2.21	1.79	3.11	2.01
DFFNet [14]	1.71	4.25	2.23	-	-	-
AANet [12]	1.99	5.39	2.55	1.80	4.93	2.32
PSMNet [17]	1.86	4.62	2.32	1.71	4.31	2.14
MANet	1.81	4.26	2.22	1.67	3.88	2.03

Explanation: The results of the proposed MANet algorithm can be seen from Table 3, where “ALL” represents all pixels in the computed image and “NOC” represents the pixels in the unoccluded regions. “D1-bg” denotes the mismatch rate in the background region, “D1-fg” represents the mismatch rate in the foreground region, and “D1-all” indicates the overall pixel mismatch rate in the image.

**Table 4:** Comparison of KITTI2012 test results.

	>2 px (%)		>3 px (%)		>5 px (%)		Mean Error (px)	
Algorithm	NOC	ALL	NOC	ALL	ALL	NOC	ALL	NOC
SGMNet [10]	3.60	5.15	2.29	3.50	1.60	2.36	0.7	0.9
DispNetC [11]	7.38	8.11	4.11	4.65	2.05	2.39	0.9	1.0
GCNet [18]	2.71	3.46	1.77	2.30	1.12	1.46	0.6	0.7
SegStereo [19]	2.66	3.19	1.68	2.03	1.00	1.21	0.5	0.6
AANet [12]	2.30	2.96	1.55	2.04	0.98	1.30	0.4	0.5
PSMNet [17]	2.44	3.01	1.49	1.89	0.90	1.15	0.5	0.6
MANet	2.26	2.87	1.39	1.82	0.83	1.09	0.5	0.5

Explanation: In the table, “>2, 3, 5 px” represents the error pixel thresholds set at 2, 3, and 5, respectively, during prediction. “Mean Error” indicates the average error of the thresholds. “NOC” represents the calculation of non-occluded pixels in the image, while “ALL” represents the calculation of all pixels in the image.

**Table 5:** Comparison of KITTI2012 test results in reflective regions.

	>2 px (%)		>3 px (%)		>5 px (%)		Mean Error (px)	
Algorithm	NOC	ALL	NOC	ALL	NOC	ALL	NOC	ALL
SGMNet [10]	22.09	25.70	15.31	18.97	10.39	13.55	3.0	3.8
DispNetC [11]	24.13	26.54	16.04	18.15	8.39	9.88	2.1	2.3
GCNet [18]	16.58	19.07	10.80	12.80	6.59	7.99	1.8	2.0
ACVNet [22]	11.42	13.53	7.03	8.67	4.14	5.20	1.4	1.5
AANet [12]	15.89	17.87	10.51	11.97	6.25	7.02	1.7	1.8
PSMNet [17]	13.77	16.06	8.36	10.18	4.58	5.64	1.4	1.6
MANet	11.93	14.32	6.86	8.70	3.61	4.75	1.3	1.5

Explanation: In the table, “>2, 3, 5 px” represents the error pixel thresholds set at 2, 3, and 5, respectively, during prediction. “Mean Error” indicates the average error of the thresholds. “NOC” represents the calculation of non-occluded pixels in the image, while “ALL” represents the calculation of all pixels in the image.

## Data Availability

We used three public datasets: SceneFlow, KITTI2015, and KITTI2012. SceneFlow: https://lmb.informatik.uni-freiburg.de/resources/datasets/SceneFlowDatasets.en.html (accessed on 27 September 2023). KITTI2015: http://www.cvlibs.net/datasets/kitti/eval_scene_flow.php?benchmark=stereo (accessed on 27 September 2023). KITTI2012: http://www.cvlibs.net/datasets/kitti/eval_stereo_flow.php?benchmark=stereo (accessed on 27 September 2023).
